# Evaluation of a point‐of‐care serum creatinine measurement device and the impact on diagnosis of acute kidney injury in pediatric cardiac patients: A retrospective, single center study

**DOI:** 10.1002/hsr2.143

**Published:** 2019-11-24

**Authors:** Satoshi Kimura, Tatsuo Iwasaki, Kazuyoshi Shimizu, Tomoyuki Kanazawa, Hirokazu Kawase, Naohiro Shioji, Yasutoshi Kuroe, Satoshi Isoyama, Hiroshi Morimatsu

**Affiliations:** ^1^ Department of Anesthesiology and Resuscitation Okayama University Hospital Okayama Japan

**Keywords:** acute kidney injury, cardiac surgical procedures, children, creatinine, point‐of‐care

## Abstract

**Background and aims:**

Agreement between measurements of creatinine concentrations using point‐of‐care (POC) devices and measurements conducted in a standard central laboratory is unclear for pediatric patients. Our objectives were (a) to assess the agreement for pediatric patients and (b) to compare the incidence of postoperative acute kidney injury (AKI) according to the two methods.

**Methods:**

This retrospective, single‐center study included patients under 18 years of age who underwent cardiac surgery and who were admitted into the pediatric intensive care unit of a tertiary teaching hospital (Okayama University Hospital, Japan) from 2013 to 2017. The primary objective was to assess the correlation and the agreement between measurements of creatinine concentrations by a Radiometer blood gas analyzer (Cre_gas_) and those conducted in a central laboratory (Cre_lab_). The secondary objective was to compare the incidence of postoperative AKI between the two methods based on Kidney Disease Improving Global Outcomes (KDIGO) criteria.

**Results:**

We analyzed the results of 1404 paired creatinine measurements from 498 patients, whose median age was 14 months old (interquartile range [IQR] 3, 49). The Pearson correlation coefficient of Cre_gas_ vs Cre_lab_ was 0.968 (95% confidence interval [CI], 0.965‐0.972, *P* < 0.001). The median bias between Cre_gas_ and Cre_lab_ was 0.02 (IQR ‐0.02, 0.05) mg/dL. While 199 patients (40.0%) were diagnosed as having postoperative AKI based on Cre_lab_, 357 patients (71.7%) were diagnosed as having postoperative AKI based on Cre_gas_ (Kappa = 0.39, 95% CI, 0.33‐0.46). In a subgroup analysis of patients whose Cre_gas_ and Cre_lab_ were measured within 1 hour, similar percentage of patients were diagnosed as having postoperative AKI based on Cre_gas_ and Cre_lab_ (42.8% vs 46.0%; Kappa = 0.76, 95% CI, 0.68‐0.84).

**Conclusion:**

There was an excellent correlation between Cre_gas_ and Cre_lab_ in pediatric patients. Although more patients were diagnosed as having postoperative AKI based on Cre_gas_ than based on Cre_lab_, paired measurements with a short time gap showed good agreement on AKI diagnosis.

## 1 INTRODUCTION

Point‐of‐care (POC) creatinine devices are capable of providing a quantitative estimation of a patient's serum creatinine concentration.^1^ POC devices, including blood gas analyzers^2^, ^3^, deliver quick results compared with a standard central laboratory, saving time and providing easy access to information to clinicians, especially in intensive care unit (ICU) settings. Some of the blood gas analyzers currently on the market can provide information on not only creatinine concentration but also biochemical, hematological, and acid‐base parameters.^2^, ^3^ The creatinine sensor used in a Radiometer blood gas analyzer (ABL 800, Radiometer Co, Copenhagen, Denmark) follows the international recommendations by the National Kidney Disease Education Program (NKDEP) Laboratory Working Group^4^ and has been shown to be a whole blood creatinine measurement method with calibration traceability to an isotope dilution mass spectometry (IDMS) standard.^3^

Plasma creatinine concentration is proportional to the muscle volume, and the values at different time points soon after birth have different reference intervals compared with those in adults.^5^ Furthermore, the required volume of a blood sample for a blood gas analyzer is only of 0.2 to 0.3 mL for neonates, which might cause errors in measurements. While some studies have shown good agreement between creatinine measurements using a POC device and measurements conducted in a central laboratory,^1^, ^2^, ^3^ there have been no studies in which the agreement between measurements of serum creatinine concentrations by a POC analyzer and those conducted in a standard laboratory have been assessed for teenage or younger patients.

The rapid simultaneous measurements of creatinine, electrolytes, and acid‐base parameters using a blood gas analyzer enable quick treatment and could change clinical practice, especially in a critically ill setting. We hypothesized that there had been more frequent creatinine measurements by a POC device in our pediatric intensive care unit (PICU) for postoperative patients than measurements in the central laboratory due to the easy access to the machine and the large range of information that can be obtained by a POC blood gas analyzer. Since worldwide guidelines such as Kidney Disease Improving Global Outcomes (KDIGO) consensus criteria^6^ do not mention appropriate frequency of creatinine measurements for diagnosis of postoperative acute kidney injury (AKI),^6^ numerous studies assessing postoperative AKI have adopted their own frequency of creatinine measurements within certain period of time after surgery.^7^, ^8^, ^9^, ^10^ If there are indeed more measurements by a POC device, these might provide additional opportunities to detect pathological changes in serum creatinine concentration, potentially improving the detection of AKI. In this sense, pediatric cardiac patients would be a good population in which to test this, because of the reported high incidence of postoperative AKI, especially after the use of cardiopulmonary bypass (CPB).^11^ Our objectives were (a) to assess the correlation and the agreement between measurements of serum creatinine concentrations by a blood gas machine and those conducted in a central laboratory for pediatric patients and (b) to compare the incidence of postoperative AKI after cardiac surgery according to serum creatinine measurements by the POC device to that based on serum creatinine measurements in the central laboratory.

## 2 MATERIALS AND METHODS

### 2.1 Design

We conducted a single‐center, retrospective study that was approved by the Okayama University Hospital Ethics Committee. The committee waived the need for informed consent for studies involving the use of the hospital's database. All regulations and measures of ethics and confidentiality were handled in accordance with the Declaration of Helsinki.

### 2.2 Study population

This was a retrospective study of patients who underwent cardiac surgery with CPB in a tertiary teaching hospital (Okayama University Hospital, Japan) and were admitted to the PICU in the hospital during the period from December 2013 to January 2017. We included patients who were 18 years of age or younger and who were admitted to the PICU for the first time. Patients without data for initial and/or postoperative serum creatinine concentrations for the first 48 hours were excluded.

### 2.3 POC creatinine testing and laboratory measurements

Blood samples were collected in standard prepared, heparinized blood gas syringes, and measurements were conducted without delay using a blood gas analyzer (ABL 800, 13B2X00079000003, Radiometer Co, Copenhagen, Denmark). The analyzer measured whole blood samples at 37°C. The laboratory in the hospital complies with standards of the National Association of Testing Authorities. The measurement of creatinine concentration by the blood gas analyzer (Cre_gas_) is based on an enzymatic method, which uses an amperometric biosensor based on enzymatic conversion.^12^ During the study period, blood gas analyses were performed at the discretion of intensivists or trained nursing staff in the PICU.

Perioperative serum creatinine concentration was also measured in a central laboratory (Cre_lab_). Blood samples were taken for laboratory analysis, sent to the laboratory within 30 minutes, and analyzed immediately. Creatinine measurement by the central laboratory is based on an enzymatic method (BM8040, CA‐Z13055TIJ, Japan Electron Optics Laboratory, Tokyo, Japan). The time of each analysis was recorded on our electronic medical chart.

### 2.4 Primary and secondary objectives

The primary objective was the correlation and the agreement between Cre_gas_ and Cre_lab_. Paired creatinine concentrations of Cre_gas_ and Cre_lab_ during a 5‐day period after surgery for which the timings of the two measurements were the closest, and the time gap of their measurements was within 1 hour, considering recommended time gap of the two measurements from the same blood sample in our institution, were investigated. The secondary objective was the agreement between the incidence of postoperative AKI based on Cre_gas_ and the incidence of postoperative AKI based on Cre_lab_ during the first 48 hours after surgery. AKI was diagnosed and classified by KDIGO criteria.^6^ According to this classification and staging system of AKI, serum creatinine concentration greater than or equal to 1.5 times the baseline or increase in serum creatinine greater than or equal to 0.3 mg/dL from the baseline creatinine concentration (both within 48 hours after surgery) constitutes “stage 1”; serum creatinine concentration greater than or equal to 2.0 times the baseline constitutes “stage 2”; and serum creatinine concentration greater than or equal to 3.0 times the baseline or initiation of renal replacement therapy constitutes “stage 3.”

### 2.5 Statistical analysis

Data are presented as n (percentages) or median (interquartile range [IQR]; 25% quartile, 75% quartile) as appropriate. The correlations between the two determination methods (Cre_gas_ vs Cre_lab_) were assessed by Pearson correlation coefficients (*r*) with 95% confidence interval (CI) and by linear regression. The linear regression is presented as a scatter plot, regression equation, and correlation coefficient. An issue of nonindependence from repeated measurements was solved by averaging the repeated measures data for each participant in sensitivity analyses. Additionally, the agreement between the two methods was analyzed, and Bland‐Altman plots were made to visualize the agreement. The Bland‐Altman plot shows the deviation between the two methods with different Cre_lab_ concentrations. The agreement between the two methods on the diagnosis of AKI was analyzed by the Kappa statistic. The baseline creatinine level and postoperative Cre_gas_ and Cre_lab_ were investigated among patients with a discordant AKI diagnosis to understand their characteristics. In order to assess the agreement between the two methods with the same number of measurements and with a short time gap, a subgroup analysis was performed for patients who had both Cre_gas_ and Cre_lab_ where the time gap between the measurements was within 1 hour. All statistical tests were two‐sided, and the significance level was defined as a *P* value of less than.05. All statistical analyses were performed using R 3.6.0 (R foundation for Statistical Computing, Vienna, Austria).

## 3 RESULTS

### 3.1 Comparison of serum creatinine concentration measured by the blood gas analyzer and that measured in the central laboratory

**Figure 1:**
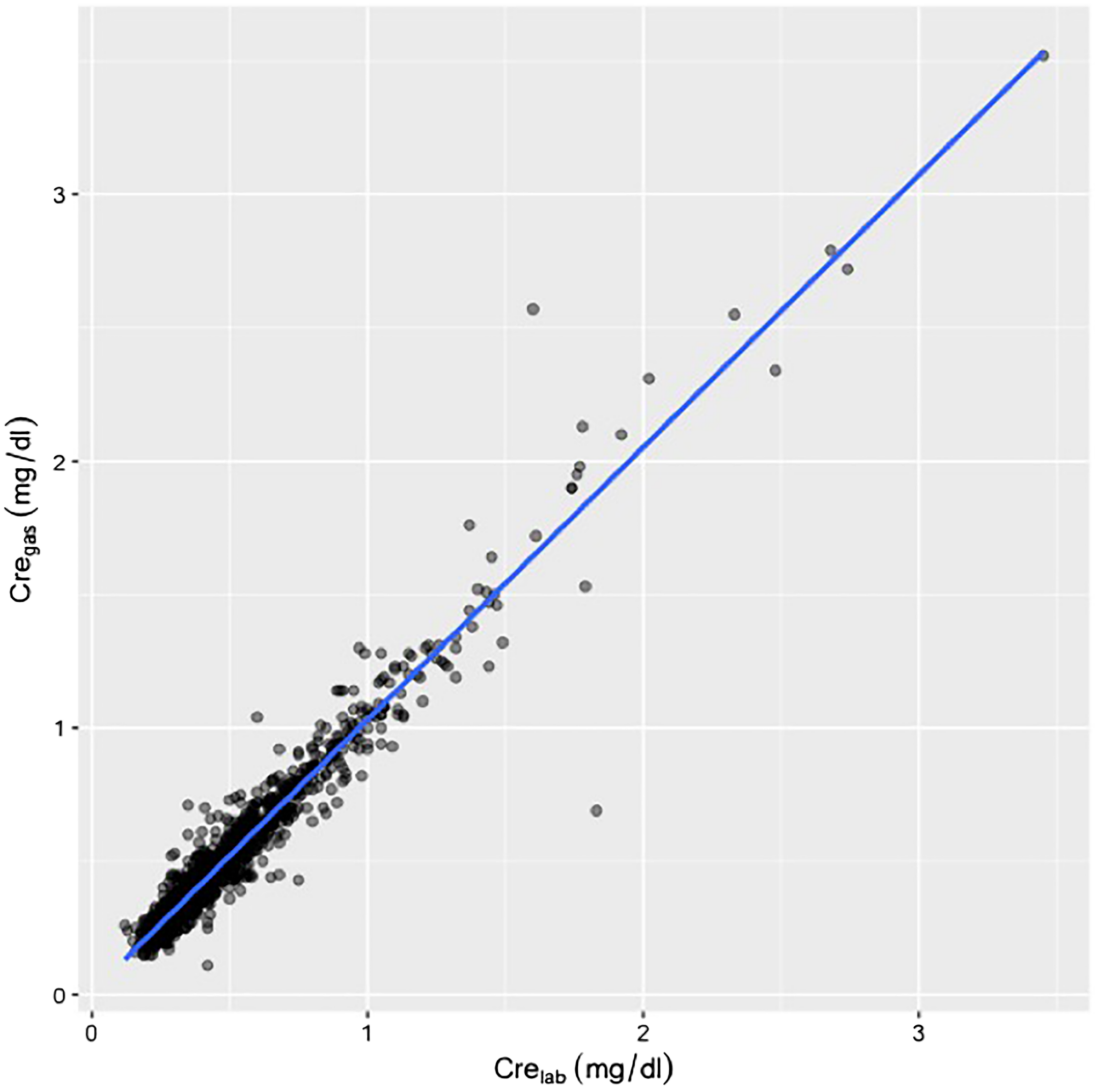
Scatter plots, regression analysis, and correlation coefficients for creatinine measurements between the blood gas analyzer and central laboratory. The result of the linear regression analysis shows the relationship: Cre_gas_ = 1.021 × Cre_lab_ + 0.010. Abbreviations: Cre_gas_, creatinine measuements by blood gas analyzer; Cre_lab_, creaninine measurements conducted in central laboratory

**Figure 2:**
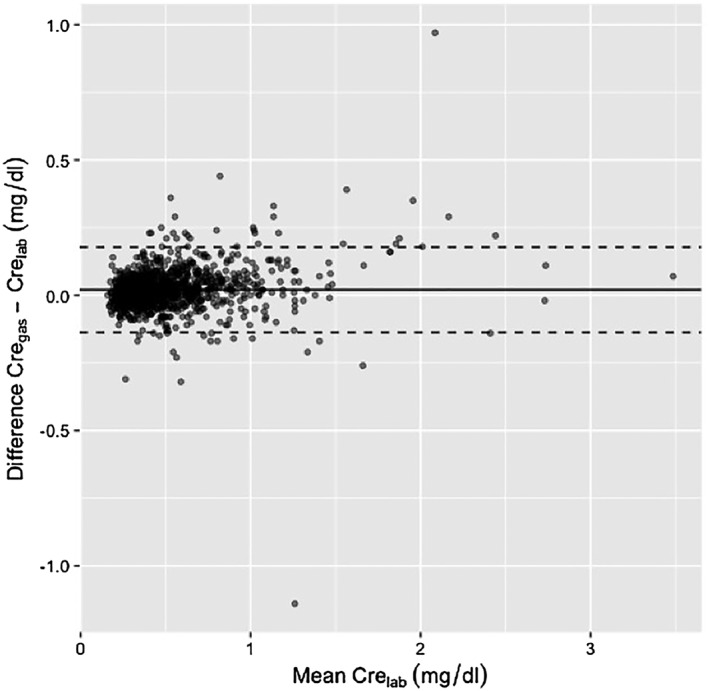
Bland‐Altman plot for the differences between blood gas analyzer serum creatinine and central laboratory serum creatinine. The 95% limit of agreement was −0.137 to 0.178 mg/dL. Abbreviations: Cre_gas_, creatinine measurements by blood gas analyzer; Cre_lab_, creaninine measurements conducted in central laboratory

A total of 556 patients were considered for the study. Fifty‐eight patients without data for initial and/or postoperative serum creatinine concentrations were excluded, and thus, a total of 498 patients were included in the study. The median age was 14 (IQR 3, 49) months, and there were 266 males (53.4%). There were 1404 paired creatinine measurements of Cre_gas_ and Cre_lab,_ with the median time gap being 25 (IQR 11, 42) minutes. The Pearson correlation coefficient of Cre_gas_ vs Cre_lab_ was 0.968 (95% CI, 0.965‐0.972, *P* < 0.001). Figure 1 shows the results of the linear regression analysis between Cre_gas_ and Cre_lab,_ with the following relationship: Cre_gas_ = 1.021 × Cre_lab_ + 0.010. After averaging the repeated measures data for each participant, the Pearson correlation coefficient of Cre_gas_ vs Cre_lab_ was 0.975 (95% CI, 0.971‐0.979), and the linear regression analysis between Cre_gas_ and Cre_lab_ was Cre_gas_ = 1.025 × Cre_lab_ + 0.010. A Bland‐Altman plot of Cre_gas_ vs Cre_lab_ is shown in Figure 2. The median bias between Cre_gas_ and Cre_lab_ was 0.02 (IQR ‐0.02, 0.05) mg/dL. The 95% limit of agreement was ‐0.137 to 0.178 mg/dL.

### 3.2 Evaluation of the impact of POC testing on the diagnosis of AKI

A total of 1439 creatinine measurements were conducted in the central laboratory within 48 hours after surgery, which meant the median number of creatinine measurements in the central laboratory was 3 (IQR 3, 3) per patient during the postoperative period. According to the KDIGO criteria based on Cre_lab_, among the 498 patients, 199 patients (40.0%) were diagnosed as having postoperative AKI. On the other hand, a total of 10 799 creatinine measurements were conducted using the blood gas analyzer, which meant the median number of creatinine measurements by the POC device was 21 (IQR 17, 26) per patient during the same period. Based on Cre_gas_, among the 498 patients, 357 patients (71.7%) were diagnosed as having postoperative AKI. While 161 patients who were diagnosed as having AKI based on Cre_gas_ were not diagnosed with AKI according to Cre_lab_, only three patients who were diagnosed with AKI based on Cre_lab_ were not diagnosed with AKI according to Cre_gas_ (Kappa = 0.39, 95% CI, 0.33‐0.46). The AKI diagnosis based on the two methods is summarized in Table 1. Among patients with a discordant AKI diagnosis, the median baseline creatinine level was 0.29 (0.25, 0.36) mg/dL, and the median difference between the maximum Cre_gas_ and the maximum Cre_lab_ within 120 hours after surgery was 0.11 (0.08, 0.15) mg/dL.

**Table 1:** AKI diagnosis based on creatinine measurements by blood gas analyzer and by those conducted in central laboratory among all of pediatric patients in the cohort (N = 498)

		Central Laboratory		
		AKI (+)	AKI (−)	
Blood gas analyzer	AKI (+)	196	161	357
	AKI (−)	3	138	141
		199	299	498

Abbreviations: AKI, acute kidney injury.

In a subgroup analysis, there were 250 patients who had both Cre_gas_ and Cre_lab_ for the first 48 hours after surgery where the time gap between the measurements was within 1 hour. The median age was 14 (IQR 3, 49) months, and there were 142 males (56.8%). Among those patients, 107 patients (42.8%) were diagnosed as having postoperative AKI based on Cre_lab_. On the other hand, based on Cre_gas_, among the 250 patients, 115 patients (46.0%) were diagnosed as having postoperative AKI. While 19 patients who were diagnosed as having AKI based on Cre_gas_ were not diagnosed with AKI according to Cre_lab_, 11 patients who were diagnosed with AKI based on Cre_lab_ were not diagnosed with AKI according to Cre_gas_ (Kappa = 0.76, 95% CI, 0.68‐0.84). AKI diagnosis based on the two methods is summarized in Table 2.

**Table 2:** AKI diagnosis based on creatinine measurements by blood gas analyzer and by those conducted in central laboratory among patients who had both Cre_gas_ and Cre_lab_ where the time gap between the measurements was within 1 h (N = 250)

		Central Laboratory		
		AKI (+)	AKI (−)	
Blood gas analyzer	AKI (+)	96	19	115
	AKI (−)	11	124	135
		107	143	250

Abbreviations: AKI, acute kidney injury; Cre_gas_, creatinine measurements by blood gas analyzer; Cre_lab_, creaninine measurements conducted in central laboratory.

## 4 DISCUSSION

### 4.1 Key findings

We found an excellent correlation between Cre_gas_ and Cre_lab_ for measuring creatinine, with *r* = .968 (95% CI, 0.965‐0.972, *P* < 0.001) and median bias of 0.02 (IQR ‐0.02, 0.05) mg/dL. The 95% limit of agreement was ‐0.137 to 0.178 mg/dL in this group of pediatric patients (younger than 18 years of age). Serum creatinine was measured about seven times more frequently with the blood gas analyzer after cardiac surgery than in the central laboratory. Despite the excellent agreement between the two methods, using Cre_gas_ and Cre_lab_, a discordant diagnosis of AKI was found in 164 patients (32.9%) (Kappa = 0.39, 95% CI, 0.33‐0.46). About 70% of the patients were diagnosed as having AKI based on Cre_gas_ and that percentage was about two times higher than the percentage of patients diagnosed as having AKI based on Cre_lab_. In a subgroup analysis of patients whose Cre_gas_ and Cre_lab_ were measured within 1 hour, similar percentage of patients were diagnosed as having postoperative AKI based on Cre_gas_ and Cre_lab_ (42.8% vs 46.0%; Kappa = 0.76, 95% CI, 0.68‐0.84).

### 4.2 Comparison with prior studies

There have been five previous studies in which the accuracy and/or the precision of POC devices for estimation of creatinine using Radiometer ABL have been assessed. In a retrospective study conducted on 650 samples from 122 critically ill patients, the accuracy of measurements by the Radiometer was assessed by comparing them with standard clinical laboratory measurements, and it was shown that the POC measurements correlated well with central pathology results (*r* ^2^ = 0.991, *P* < 0.001).^13^ Bloomed et al prospectively assessed a total of 183 pairs of samples for creatinine from 207 patients and showed that the mean difference was +0.018 mg/dL and that the 95% limit of agreement^2^ was ‐0.18 to 0.21 mg/dL. In another prospective cohort study, of 250 samples from 82 critically ill patients, the mean difference between serum creatinine measured by a central laboratory and that measured by POC testing was +0.11 mg/dL (95% limits of agreement: ‐0.13 to +0.34 mg/dL).^14^ Skurup et al also measured creatinine concentrations, in a total of 104 serum samples, and assessed the performance of the creatinine sensor of an ABL blood gas analyzer.^3^ They found excellent agreement of POC creatinine assays with laboratory creatinine assays using IDMS calibration. While our study was the first to assess the accuracy of measurements by this POC device exclusively for patients younger than 18 years of age, our results are consistent with those of previous studies, showing acceptable agreement between creatinine measurements by a blood gas analyzer and by laboratory assays.

The impact of POC creatinine testing on clinical practice, however, is unclear. Although the shortened average time to diagnosis has been evaluated by using various POC devices in some studies,^15^, ^16^ there has been no study on the impact of measurements of creatinine by a blood gas analyzer, especially on the detection of AKI in patients. We showed that at our hospital, the frequency of creatinine concentration measurements by a POC analyzer was about seven times higher than that of creatinine concentrations measurements in a central laboratory. In addition, we found that the number of patients diagnosed as having AKI by Cre_gas_ was about two times greater than the number of patients diagnosed as having AKI by Cre_lab_, and discordant diagnosis of AKI was found in around one‐third of patients regardless of the good agreement between the two methods for creatinine concentration determination.

### 4.3 Interpretation

According to the US Clinical Laboratory Improvement Amendments (USCLIA), the criteria for acceptable performance of measurements is the target value ±0.3 mg/dL for creatinine.^17^ We demonstrated high levels of agreement, with a low median difference of Cre_gas_ and Cre_lab_ and 95% limit of agreement within the range specified by the USCLIA. Our study, which is the first one to assess the performance of a POC device exclusively on patients younger than 18 years of age, supports the clinical usefulness of Cre_gas_ for patients with a wide range of ages, when taken together with the results of the other studies mentioned above.

The reason for serum creatinine being measured much more frequently by the POC device than in the central laboratory might be related to the immediate availability of the results.^15^, ^16^ Clinicians might use the POC device due to the ease of access in clinical practice. The blood gas data obtained by the analyzer, in addition to that on creatinine levels, could be another reason for the high frequency of measurements. Clinicians might perform more POC tests in order to check the acid‐base status for each patient rather than to check Cre_gas_. Additional studies are needed to understand clinicians' preferences and choices regarding the use of POC devices for creatinine measurements.

Despite the excellent agreement between the two methods and the potential advantage of easy access and frequent information by the POC device, we need careful discussion before using the POC device frequently for diagnosis of AKI based on KDIGO criteria for pediatric patients because of the discordant diagnosis of AKI between the two methods, as shown in our study. There are several possible explanations for more patients being diagnosed with AKI based on Cre_gas_ than based on Cre_lab_ and the frequent discordant diagnosis of AKI despite good correlation between the two methods. One possibility is superior detection of increased creatinine concentration by multiple Cre_gas_ measurements. The more frequently creatinine is measured, the more possible it becomes for measurement to show the timing of creatinine increase. The average number of creatinine measurements in the central laboratory was only three per patient within 48 hours after surgery, which was only one‐seventh of the average number of measurements by the blood gas analyzer. This means that Cre_lab_ might miss the chance for detecting a postoperative increase in creatinine concentration.

A second possible explanation for the discordant diagnosis of AKI despite good correlation between the two methods is due to a potential error of each Cre_gas_ measurement. Although our study showed a high level of agreement of Cre_gas_, we could still see some differences between Cre_gas_ and Cre_lab_, as shown in Figure 1. In addition, although the reported precision of the Radiometer ABL is relatively high,^1^, ^3^ there is a risk of obtaining incorrect creatinine concentration when the concentrations are measured many times by a blood gas analyzer. Increased risk of errors due to multiple measurements of Cre_gas_ could be one reason. Furthermore, low creatinine concentration in pediatric patients should also be considered in the discordant diagnosis of AKI between the two method. As the KDIGO criteria uses baseline creatinine concentration for reference and the multiplication of the baseline measurement for AKI diagnosis, low baseline creatinine concentration would lower the cutoff of creatinine concentration for AKI diagnosis, such that a small error would have a big impact on the diagnosis of AKI. For example, a patient with a baseline creatinine concentration of 0.8 mg/dL would be diagnosed as AKI stage 1 with postoperative creatinine concentration 1.2 mg/dL. If a patient has a baseline creatinine concentration of 0.3 mg/dL, they would be diagnosed as AKI stage 1 with postoperative creatinine concentration 0.45 mg/dL, which is just 0.15 mg/dL higher than the baseline value and could easily happen due to a measurement error. In a pediatric population where the baseline creatinine concentration is relatively low and small variability could have a large influence on AKI diagnosis, increased risk of errors due to multiple measurements could explain the high AKI rate based on Cre_gas_. Indeed, among patients with disagreement on AKI diagnosis in our study, the median baseline creatinine level was as low as 0.29 mg/dL.

It is difficult to determine which explanation is more likely based on our data. However, our subgroup analysis showed that the rates of AKI diagnosis based on Cre_gas_ and Cre_lab_ were similar among patients whose Cre_gas_ and Cre_lab_ were measured within 1 hour, along with a high Kappa value. This finding supports both the first and the second explanations for more patients being diagnosed with AKI based on Cre_gas_ than based on Cre_lab_ despite the good correlation between the two methods. Additional studies are needed to show the utility of frequent creatinine measurements by POC devices for diagnosis of AKI based on KDIGO criteria for pediatric patients.

### 4.4 Limitations/generalizability

There are several limitations in this study. First, it was a retrospective study and, thus, potentially subject to systematic error and bias. However, the clinical and electronic data were collected prospectively and were measured independently, and the data were, therefore, not subject to unintended manipulation. Second, this was a small, single‐center study with a significant chance of a type I error and weak generalizability. Third, we used pairs of creatinine measurements for which the time gap between measurements of Cre_gas_ and Cre_lab_ was within 1 hour to assess the correlation and the agreement. This is because the two samples obtained at the same time were analyzed at different places, with an associated time gap, and because our electronic records only have information on the time of analyses, not the time of blood sampling. Although changes in creatinine are usually slow and unlikely to show a large difference within such a short period, this does not reflect a perfect assessment of the accuracy of Cre_gas_. In addition, dramatic change in volume status during postoperative period might affect our findings. Those limitations make it difficult to generalize our results. Furthermore, this study did not show any patients' outcomes. The impact of high frequent diagnosis of AKI by POC device on clinical outcomes remains unclear. Considering that the cost of one blood gas measurement is about 1.2 times higher than that of one ordinal chemistry panel in the Japanese public medical system, the advantage of frequent creatinine measurements with the POC device is unclear. However, it should be noted that our objectives were to evaluate the POC serum creatinine measurement device for pediatric patients and to assess the impact on diagnosis of AKI. In this regard, a future prospective study with scheduled measurements of Cre_gas_ and Cre_lab_ from a range of patients and evaluating clinical outcomes should be conducted.

## 5 CONCLUSIONS

There was an excellent correlation between measurements of creatinine by the POC blood gas analyzer and those conducted in the central laboratory for patients who were younger than 18 years of age and underwent cardiac surgery. Although a discordant diagnosis of postoperative AKI based on KDIGO criteria was found in about one third of the pediatric patients between the two methods when using all the measurements, the paired measurements with a small time gap showed a good agreement on AKI diagnosis. Additional studies are needed to show the efficacy of frequent creatinine measurements by POC devices for diagnosis of AKI based on KDIGO criteria for pediatric patients.

## FUNDING INFORMATION

The authors received no specific funding for this work.

## CONFLICT OF INTEREST

The authors declare no conflicts of interest.

## AUTHOR CONTRIBUTIONS

Conceptualization: Satoshi Kimura, Tatsuo Iwasaki, Kazuyoshi Shimizu, Tomoyuki Kanazawa, Hirokazu Kawase, Naohiro Shioji, Yasutoshi Kuroe, Satoshi Isoyama, Hiroshi Morimatsu

Formal Analysis: Satoshi Kimura

Investigation: Satoshi Kimura, Tatsuo Iwasaki, Kazuyoshi Shimizu, Tomoyuki Kanazawa, Hirokazu Kawase, Naohiro Shioji, Yasutoshi Kuroe, Satoshi Isoyama

Methodology: Satoshi Kimura, Tatsuo Iwasaki, Kazuyoshi Shimizu, Tomoyuki Kanazawa, Hirokazu Kawase, Naohiro Shioji, Yasutoshi Kuroe, Satoshi Isoyama, Hiroshi Morimatsu

Supervision: Tatsuo Iwasaki, Hiroshi Morimatsu

Writing—Original Draft Preparation: Satoshi Kimura

Writing—Review and Editing: Satoshi Kimura, Tatsuo Iwasaki, Kazuyoshi Shimizu, Tomoyuki Kanazawa, Hirokazu Kawase, Naohiro Shioji, Yasutoshi Kuroe, Satoshi Isoyama, Hiroshi Morimatsu

All authors have read and approved the final version of the manuscript.

Satoshi Kimura had full access to all of the data in this study and takes complete responsibility for the integrity of the data and the accuracy of the data analysis.

## TRANSPARENCY STATEMENT

Satoshi Kimura affirms that this manuscript is an honest, accurate, and transparent account of the study being reported that no important aspects of the study have been omitted and that any discrepancies from the study as planned (and, if relevant, registered) have been explained.

## DATA SHARING STATEMENT

No original data are shared because of no informed consent for data sharing.
